# Supplementary material for: Trapped in my inner prison—Cross-sectional examination of internal and external entrapment, hopelessness and suicidal ideation
Source: PLoS One. 2022 Jul 19;17(7):e0270985. doi: 10.1371/journal.pone.0270985 (PMC9295950; doi:10.1371/journal.pone.0270985)
Supplement: S1 File — (DOCX) [file pone.0270985.s001.docx]

| **Table 1** |  |  |
| --- | --- | --- |
| *Sociodemographic Characteristics of the Participants* | | |
| Baseline characteristics | *n* | % |
| Gender |  |  |
| Female | 342 | 75 |
| Male | 111 | 24 |
| Non-binary | 1 | < 1 |
| Country of Origin |  |  |
| Germany | 414 | 91 |
| Austria | 15 | 3 |
| Switzerland | 9 | 2 |
| Other | 16 | 3 |
| Relationship status |  |  |
| In a relationship | 272 | 60 |
| Single | 182 | 40 |
| Marital status |  |  |
| Unmarried/single | 371 | 82 |
| Married | 67 | 15 |
| Divorced | 14 | 3 |
| Widowed | 2 | < 1 |
| Highest educational level |  |  |
| No school-leaving certificate/not graduated | 1 | < 1 |
| Still attending school | 8 | 2 |
| Lower secundary school certificate | 4 | < 1 |
| Secundary school certificate | 32 | 7 |
| Vocational school | 20 | 4 |
| Vocational baccalaureate diploma | 34 | 8 |
| A-level/General matriculation standard | 187 | 41 |
| Other | 168 | 37 |
| Vocational/professional qualification |  |  |
| No further qualification | 136 | 30 |
| Apprenticeship/internship | 83 | 18 |
| University/college | 180 | 40 |
| Other | 55 | 12 |
| Risk factors for suicidal thoughts/behaviour |  |  |
| Past mental disorder | 167 | 37 |
| Current mental disorder | 138 | 30 |
| Past suicide attempt | 42 | 9 |
| Seriousness in the last suicide attempt | 23 | 5 |
| Suicidal ideation in the past two weeks | 98 | 22 |

**Table 2. Additional descriptive statistics and Pearson Product-Moment-Correlations**

|  | *M* | *SD* | *SE* | Range | Med. | Skew. | Kurt. | 1. IE | 2. EE | 3. BHS | 4. BSS-S |
| --- | --- | --- | --- | --- | --- | --- | --- | --- | --- | --- | --- |
| 1. IE | 8.36 | 8.28 | .39 | 0-24 | 6 | .59 | -1.10 | ˗ |  |  |  |
| 2. EE | 13.17 | 9.81 | .46 | 0-40 | 11 | .49 | -.81 | .85^**^ | ˗ |  |  |
| 3. BHS | 6.52 | 5.61 | .26 | 0-20 | 4 | .84 | -.55 | .73^**^ | .68^**^ | ˗ |  |
| 4. BSS-S | 1.19 | 2.23 | .10 | 0-10 | 0 | 2.05 | 3.58 | .63^**^ | .56^**^ | .72^**^ | ˗ |
| 5. Total-E | 21.54 | 17.41 | .82 | 0-64 | 17 | .49 | -1.04 | .96^**^ | .97^**^ | .73^**^ | .62^**^ |
| *Note.* IE = Internal Entrapment Subscale; EE = External Entrapment Subscale; BHS = Beck-Hopelessness-Scale; BSS-S = Beck-Suicidal Ideation-Scale-Screen; Total-E = (total) Entrapment Scale; *M* = mean; *SD* = standard deviation; *SE* = standard error; Med. = Median; Skew. = skewness of the distribution; Kurt. = kurtosis of the distribution.***p* < .01. | | | | | | | | | | | |

**Fig 1. Additional Mediation model 3 with total entrapment as predictor.**


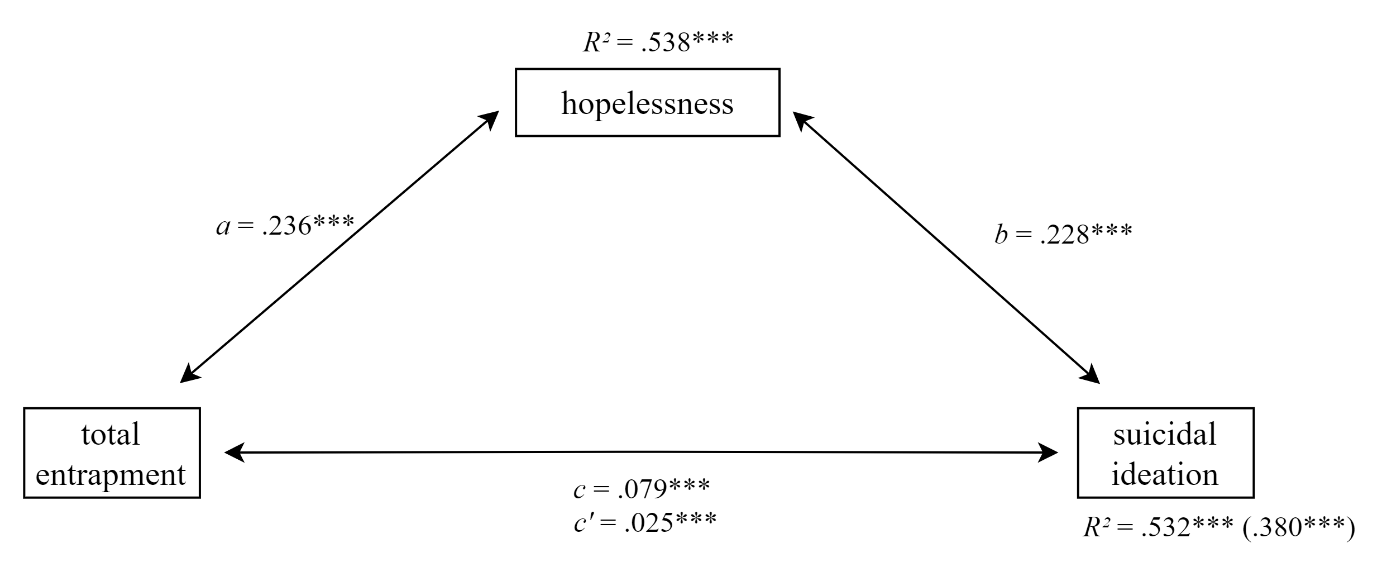


**Table 3. Total, direct and indirect effects of all mediation models.**

|  | Effect | *SE* | 95% CI | *CS* |
| --- | --- | --- | --- | --- |
| Mediation model 1 |  |  |  |  |
| Total effect | .170 | .013 | [0.145, 0.195] | .631 |
| Direct effect | .062 | .014 | [0.034, 0.090] | .230 |
| Indirect effect | .108 | .015 | [0.081, 0.138] | .402 |
| Mediation model 2 |  |  |  |  |
| Total effect | .127 | .010 | [0.107, 0.148] | .561 |
| Direct effect | .030 | .011 | [0.008, 0.052] | .132 |
| Indirect effect | .097 | .012 | [0.076, 0.122] | .429 |
| Mediation model 3 |  |  |  |  |
| Total effect | .079 | .006 | [0.067, 0.090] | .616 |
| Direct effect | .025 | .007 | [0.012, 0.039] | .196 |
| Indirect effect | .054 | .007 | [0.041, 0.069] | .421 |

*Note.* Mediation model 1: internal entrapment (*X*), hopelessness (*M*), suicidal ideation (*Y*); mediation model 2: external entrapment (*X*), hopelessness (*M*), suicidal ideation (*Y*); mediation model 3: (total) entrapment (*X*), hopelessness (*M*), suicidal ideation (*Y*); *SE* = standard estimation error; 95% CI = 95% confidence interval; *CS* = complete standardized effect.

**Table** **4. Regression coefficients for the prediction of suicidal ideation.**

| Predictor |  | Predicted Variables | | | | | | |
| --- | --- | --- | --- | --- | --- | --- | --- | --- |
|  |  | *M* (BHS) | | |  | *Y* (BSS) | | |
|  |  | Coefficient | *SE* | *p* |  | Coefficient | *SE* | *p* |
| *X* (IE) | *a* | .496 | .023 | < .001 | *c* | .170 | .013 | < .001 |
|  |  | *R²* = .534 | | |  | *R²* = .398 | | |
|  |  | *F* (1, 452) = 462.826, *p* < .001 | | |  | *F* (1, 452) = 181.494, *p* < .001 | | |
|  |  |  |  |  | *c'* | .062 | .014 | < .001 |
| *M* (BHS) |  | ˗ | ˗ | ˗ | *b* | .218 | .026 | < .001 |
| Constant | *i1* | 2.376 | .202 | < .001 | *i2* | -.744 | .078 | < .001 |
| *X* + *M* |  |  |  |  |  | *R²* = .539 | | |
|  | |  |  |  |  | *F* (2, 451) = 130,088, *p* < .001 | | |
| *X* (EE) | *a* | .392 | .019 | < .001 | *c* | .127 | .010 | < .001 |
|  |  | *R*² = .468 | | |  | *R*² = .315 | | |
|  |  | *F* (1, 452) = 425.563, *p* < .001 | | |  | *F* (1, 452) = 150.183, *p* < .001 | | |
|  |  |  |  |  | *c'* | .030 | .011 | .008 |
| *M* (BHS) |  | ˗ | ˗ | ˗ | *b* | .249 | .026 | < .001 |
| Constant | *i1* | 1.363 | .240 | < .001 | *i2* | -.823 | .089 | < .001 |
| *X* + *M* |  |  |  |  |  | *R²* = .523 | | |
|  |  |  | | |  | *F* (2, 451) = 120.997, *p* < .001 | | |
| *X* (E) | *a* | .236 | .010 | < .001 | *c* | .079 | .006 | < .001 |
|  |  | *R*² = .538 | | |  | *R*² = .380 | | |
|  |  | *F* (1, 452) = 532.243, *p* < .001 | | |  | *F* (1, 452) = 181.867, *p* < .001 | | |
|  |  |  |  |  | *c'* | .025 | .007 | < .001 |
| *M* (BHS) |  | ˗ | ˗ | ˗ | *b* | .228 | .027 | < .001 |
| Constant | *i1* | 1.428 | .221 | < .001 | *i2* | -.829 | .082 | < .001 |
| *X* + *M* |  |  |  |  |  | *R²* = .532 | | |
|  |  |  | | |  | *F* (2, 451) = 128.182, *p* < .001 | | |

*Note.* IE = internal entrapment subscale; EE = external entrapment subscale; E = (total) entrapment scale; BHS = Beck-Hopelessness-Scale; BSS-S = Beck-Suicidal Ideation-Scale-Screen.

**Additional Results**

Regarding to the annotations of the reviewers, correlation analysis and a mediation model including (total) entrapment as a predictor were conducted post hoc.

The results of the correlation analysis shows that all constructs, including (total) entrapment, were significantly and highly correlated with each other (*p* < .01).

As in the preceding mediation models, all coefficients were significant (*p* < .001). An effect of entrapment on suicidal ideation was observed (*c* = .079, *SE* = .006). After entering the mediator into the model, entrapment predicted hopelessness (*a* = .236, *SE* = .010), which in turn predicted suicidal ideation (*b* = .228, *SE* = .027). The effect from entrapment on suicidal ideation was reduced when hopelessness was added to the model (*c‘* = .025, *SE* = .007).

Results also indicated an indirect effect from entrapment on suicidal ideation via hopelessness (ab = .054, SE = .007, 95%-CI [0.041, 0.069]). Since the confidence interval did not include zero, there was a significant mediation effect. The completely standardized effect was *CS_ab_* = .421. The explained variance in suicidal ideation changed from 38% to 53.2% when hopelessness was considered as a mediator. Besides, 53.8% of the variance in hopelessness can be explained through entrapment.

Comparing the results of total entrapment to those of the preceding analyses, the completely standardized indirect effect of (total) entrapment (*CS_ab_* = .421) was slightly smaller than the completely standardized indirect effect of external entrapment (*CS_ab_* = .429), and thus larger than the completely standardized indirect effect of internal entrapment (*CS_ab_* = .402). The variance in suicidal ideation explained by entrapment (*R²* = .380) was slightly lower than the explained variance through internal entrapment (*R²* = .398), even when hopelessness was considered (*R²* = .532 < .539). But compared to the second mediation model, the explained variance by entrapment is greater for each variable.

According to the preceding results, the association between entrapment and suicidal ideation was partially mediated by hopelessness.
