# Supplementary material for: Trapped in my inner prison—Cross-sectional examination of internal and external entrapment, hopelessness and suicidal ideation
Source: PLoS One. 2022 Jul 19;17(7):e0270985. doi: 10.1371/journal.pone.0270985 (PMC9295950; doi:10.1371/journal.pone.0270985)
Supplement: Attachment — Submitted filename: PLOS One Review.docx [file pone.0270985.s003.docx]

Thank you for the opportunity to review your manuscript! I am fascinated by the ongoing IMV debate, specifically regarding the specific constructs hypothesized to play a role in suicide phenomenology. This study makes and important contribution thereto and has interesting implications moving forward. However, while this study is standard in its methodological approach, it is also significantly limited by a cross-sectional mediation design and may benefit from the inclusion of an additional mode. Finally, there are several bold but unsubstantiated claims in the manuscript that will require further contextualization, discussion, and most importantly, citation:

General comments:

I do understand and appreciate that the authors may consider English as a second language. However, there were points where the narrative was unclear and could benefit from copyediting. I was able to understand the thrust, but re-reading and re-evaluating readability will help make the manuscript clearer for the reader.

The authors should refrain from using the term “confirmed” when referencing their own hypotheses or the results of extant literature. The nature of parametric statistics precludes the confirmation of results and only *support for* the observed results.

Background

Generally, when introducing a cited study please briefly describe the sample and the design of the cited study. This will give the reader more context for the results being described

Lines 73 – 74 will require a citation for the assertion that “research...has revealed that internal entrapment seems to be more important...”

Please briefly describe the findings of Owen, Dempsey et al., more in depth

Lines 78 – 79 appears to be an assertion that comes out of the blue; however, it becomes clearer when discussing this assertion in the Discussion section of the manuscript, lines 230 – 233. Clarifying this assertion in the Introduction would be useful

Line 97; although I understand the reflex to argue from a perspective of reason, it is not sufficient to argue for the differential effects of entrapment dimensions on hopelessness based on reason; reasonable based on...? The Cry of Pain model? Johnson, Gooding? These at least should be cited and expanded upon a bit more

Measures

Please provide a detailed breakdown of demographics in a table to further contextualize the sample; among the variables already include, likely additions should include racial identity, gender identity, and the baseline risk variables (suicide attempt, suicidal ideation, etc.)

Please provide further detail as to whether measures were sum scored or mean scored

Please provide the following estimates in the correlation table in addition to those provided: standard error (SE), skew, kurtosis, range, for each of the constructs measured

Statistical analyses

Line 165; the assertion of either full or partial mediation is contrary to the hypotheses and strikes of HARKing; please clarify here and in the hypotheses whether you predict full or partial mediation, not both.

It seems reasonable that the authors should test the hypothesized model with the Entrapment Scale total score considering their intention to determine whether internal vs. external entrapment explain more variance in SI as explained by hopelessness; this would make for a potentially stronger paper by match some of their cited literature (i.e., Littlewood, Gooding), testing an inference of the IMV, and align with their assertion that “it remains unclear how and why internal and external entrapment influence suicidal ideation”. Testing all three models would permit more nuanced discussion of theoretical implications.

Discussion

Line 263; please compare and contrast how your findings are contrary to those of the cited studies

Lines 267 – 274; these are major assumptions regarding the impact of the COVID-19 pandemic on suicidal ideation and external entrapment that will require contextualization within the available literature. For example, Bryan, Bryan, and Baker (2021) found no relationship between physical distancing and with worse mental health outcomes. This observation has implications for the relationship between the COVID-19 pandemic and external entrapment.

Line 278; given that both external and internal entrapment in relation to suicidal ideation were significantly explained by hopelessness to some extent, I am not convinced that these constructs require separation.

Their conclusions may be supported by inclusion of the proposed total score model. If a worse model fit is observed in contrast to the internal or external entrapment models, this conclusion would be significantly strengthened

Additionally, the authors may wish to discuss the findings in different contexts; for example, they may wish to reference the findings of Oakey-Frost et al., 2021 who found that internal but not external entrapment with fearlessness about death explained the relationship between PTSD and suicidal ideation. Thus, the effect of one vs. the other may vary according to important interindividual differences

Lines 279 – 278; the argument for differential treatment approaches for internal and external entrapment is potentially reasonable, but unsubstantiated with regard to approaches to external entrapment specifically.

Additionally, the question must be asked: are we aiming to improve entrapment or suicidal thoughts and behaviors? Other viable treatment options do exist and should be discussed; The Collaborative Assessment and Management of Suicidality (CAMS; Jobes, 2017) is a framework used to target entrapment OR internal vs. external entrapment as the driver of suicidal thoughts and behaviors based on the needs of the individual in question. This critique includes lines 311 – 312. Thus, the authors may be correct that internal vs. external entrapment may require different approaches based on the needs of the individual and their suicidal driver. But this claim has to be substantiated with citation and discussion.

Finally, the authors should discuss the limitations of cross-sectional mediation in the context of Maxwell & Cole, 2007.

Figure

I would prefer that arrows in the models are bidirectional. While the conclusions may be theoretically supported, inferring directionality and therefore temporal relationships is not supported in cross-sectional mediation models.

Thank you for allowing me to review your manuscript and for your valuable contribution to the suicide prevention literature base. I hope that my comments and feedback are of value to the authors and to the editor(s) of the journal. I genuinely appreciate the opportunity to engage in this valuable scientific discourse.
