# Supplementary material for: Trapped in my inner prison—Cross-sectional examination of internal and external entrapment, hopelessness and suicidal ideation
Source: PLoS One. 2022 Jul 19;17(7):e0270985. doi: 10.1371/journal.pone.0270985 (PMC9295950; doi:10.1371/journal.pone.0270985)
Supplement: Attachment — Submitted filename: Detailed_Responses_to_the_Reviewers.docx [file pone.0270985.s004.docx]

1. Please ensure that your manuscript meets PLOS ONE's style requirements, including those for file naming. The PLOS ONE style templates can be found at
   https://journals.plos.org/plosone/s/file?id=wjVg/PLOSOne_formatting_sample_main_body.pdf and 
   https://journals.plos.org/plosone/s/file?id=ba62/PLOSOne_formatting_sample_title_authors_affiliations.pdf.

***Response 1:***

*The manuscript has been adapted and now meets the PLOS ONE’s style requirements.*

*The following changes have been made:*

- *The corresponding author’s physical address was replaced by the corresponding author’s email address and initials in parentheses after the e-mail address.*
- *Headings (Level 1, 2 and 3) now meet the requirements of the font size and type.*
- *Figures now are cited as required: “Fig 1”, “Fig 2”, …*
- *The figure titles are now written in bold type.*
- *Tables are now cited as required and table titles were changed into bold print.*

2. Please provide additional details regarding participant consent. In the ethics statement in the Methods and online submission information, please ensure that you have specified what type you obtained (for instance, written or verbal, and if verbal, how it was documented and witnessed). If your study included minors, state whether you obtained consent from parents or guardians. If the need for consent was waived by the ethics committee, please include this information.

***Response 2:***

*Before starting the survey, participants were informed about the purpose of the study, the voluntary nature of participation, data storage and security, and gave their informed consent by accepting the conditions on the first page of the online survey by clicking a checkbox. In case they did not check the box, they were excluded from the study.
(The part in italics was added to the manuscript.)*

3. Thank you for stating the following financial disclosure:
    [This study did not receive funding.]

At this time, please address the following queries:

a) Please clarify the sources of funding (financial or material support) for your study. List the grants or organizations that supported your study, including funding received from your institution. 
b) State what role the funders took in the study. If the funders had no role in your study, please state: “The funders had no role in study design, data collection and analysis, decision to publish, or preparation of the manuscript.”
c) If any authors received a salary from any of your funders, please state which authors and which funders.
d) If you did not receive any funding for this study, please state: “The authors received no specific funding for this work.”

Please include your amended statements within your cover letter; we will change the online submission form on your behalf.

***Response 3:*** *We did not receive funding for this study. We added this to the cover letter as well as to the manuscript.*

4. We note that you have indicated that data from this study are available upon request. PLOS only allows data to be available upon request if there are legal or ethical restrictions on sharing data publicly. For more information on unacceptable data access restrictions, please see http://journals.plos.org/plosone/s/data-availability#loc-unacceptable-data-access-restrictions.

In your revised cover letter, please address the following prompts:

a) If there are ethical or legal restrictions on sharing a de-identified data set, please explain them in detail (e.g., data contain potentially sensitive information, data are owned by a third-party organization, etc.) and who has imposed them (e.g., an ethics committee). Please also provide contact information for a data access committee, ethics committee, or other institutional body to which data requests may be sent.

b) If there are no restrictions, please upload the minimal anonymized data set necessary to replicate your study findings as either Supporting Information files or to a stable, public repository and provide us with the relevant URLs, DOIs, or accession numbers. For a list of acceptable repositories, please see http://journals.plos.org/plosone/s/data-availability#loc-recommended-repositories.

We will update your Data Availability statement on your behalf to reflect the information you provide.

***Response 4:***

a) *There are no ethical or legal restrictions.*
b) *The data set will be provided.*
5. Your ethics statement should only appear in the Methods section of your manuscript. If your ethics statement is written in any section besides the Methods, please delete it from any other section.

***Response 5:***

*“Ethics approval and consent to participate” has been deleted from the Declaration-Section.*

**Reviewer #1**

This is an interesting study. It is limited by a non-clinical cross-sectional sample, although the authors point out these limitations.

Methods
The Sample results (p.5, l. 105-112) should be reported in the Results section.

***Response:*** *We consider this sample information necessary to provide insight into the composition of the sample at this point. The sample information is not reported in the results section because the results section should mainly highlight the results of the statistical analyses that were conducted to test our hypotheses (i.e., correlation and mediation analysis). We hope that it is okay if we leave it like that.*

More information about the sample would be useful, such as education level, socio-economic status, etc. ***Response:*** *sociodemographic characteristics are summed up in a table (Table 1 in supplementary material), which can be found in the supplementary material. Information on this table have been added to the participants’ section.*

What proportion were students recruited from the University?

***Response:*** *The proportion of students from the University of Duisburg-Essen was not measured per se but the proportion of (university) students in general (i.e., irrespective of at which university they were enrolled) was 28% (n= 125). We added this information to the participants’ section.*

Measures
How were past and current mental disorders measured?

***Response:*** *Participants were asked with single items each whether they had a mental disorder in the past or at the moment and whether they received treatment. We are aware that this is only self-report, this is why we used the wording “participants reported a mental disorder”. We added the self-report nature to the limitations.*

The scoring of the BSS is not clearly explained (sentence on p.6, l.136-137). How were the five items which were used scored?

***Response:*** *The (total) BSS score ranges from 0 to 38 and is formed from the sum of the first 19 items. The last two items (20 and 21) describe former suicide attempts (and not thoughts) and are usually not included in the total score. However, because of the ambiguous factor structure of the total BSS score, the BSS-Screen was used to describe suicidal ideation. In contrast to the total BSS-Score, the BSS-Screen score has a unidimensional factor structure. This BSS-Screen includes the sum score of only the first five items of the BSS. We added information on this to the manuscript.*

Results
In Table 1 it seems that the Min and Max are the possible min. and max. values for the scale, rather than the results obtained from the sample. These should be the min. and max. values from the sample. If the distributions were very skewed, median and interquartile range should be reported too.

***Response:*** *Thank you for this suggestion. The “Min” and “Max” were replaced by the range, so it is easier to understand that those are the values from the sample. Information on skewness, kurtosis, range and median have also been added to the table.* *We hope this meets your expectations.*

The means obtained for all the psychological measures (Table 1) are fairly low, as this is not a clinical sample. Could the authors please comment on how skewed the distributions were and whether this could have implications for the statistical methods used. Often with non-clinical samples the distributions of psychological scales are extremely skewed.

***Response:*** *We followed your suggestion above and added skewness and kurtosis to the table. Due to the large sample and the fact that bootstrapping does not depend on normal distribution, the skewness and kurtosis has not been considered to impact validity of the results any further.*

Discussion
The sentence on p. 12, l. 250-251 makes an inference about the role of internal entrapment on the development of hopelessness, however this is a cross sectional non-directional study.

***Response:*** *You are completely right, that this is a limitation and. We changed the wording and added the limitations of the study, so it now reads like this: “In this study, the large amount of explained variance in hopelessness through internal entrapment is striking and emphasizes its role in the development of hopelessness. However, it has to be considered that those were only cross-sectional results. These findings should be replicated in a clinical sample with longitudinal data.” We hope that this meets your expectations.*

Although hopelessness mediated the association between external entrapment and suicidal ideation to a slightly greater extent, the results are very similar to those of internal attachment. Because the results are so similar, and without testing for significant differences of the explained variance (R2) and the coefficients, it is difficult to draw a definitive conclusion that one mediation is stronger than the other.

***Response:*** *We agree. However, Internal and external entrapment being so similar in its effect on hopelessness / SI is still surprising considering previous findings in which internal entrapment seems to have a more important role with regard to suicidality (Höller et al., 2021; O’Connor & Portzky, 2018; Owen et al., 2018; Rasmussen et al., 2010) . Indeed, it is a limitation that there was no testing for significance of the explained variances, coefficients and standardized effects. Anyway, the completely standardized effect is a proper measure to compare effects from different mediation models (Hayes, 2017), the size of the effect itself is already a surprising and relevant result.*

Strengths and Limitations
The authors mention the use of an “attention check item” (p.14, l.294), but this is not included in the Measures section.

***Response:*** *The attention check item is already mentioned in the Methods (sample) section. We decided not to repeat this information in the measures section, as this item only served to filter out those people who completed the survey responsibly. Therefore, the attention check item is not a measurement per se. Participants who did not correctly answer the attention check item were excluded from the data analyses. We added the following sentences to the manuscript: “The attention check item asked participants to check the answer option “4”. In case they did not check this item correctly due to inattentiveness, they were excluded from analyses.” In the participants section is statet that 454 participants answered the attentiom check item correctly and completed all relevant questionnaires. We hope this answers your question.*

**Reviewer #2**

Thank you for the opportunity to review your manuscript! I am fascinated by the ongoing IMV debate, specifically regarding the specific constructs hypothesized to play a role in suicide phenomenology. This study makes and important contribution thereto and has interesting implications moving forward. However, while this study is standard in its methodological approach, it is also significantly limited by a cross-sectional mediation design and may benefit from the inclusion of an additional mode. Finally, there are several bold but unsubstantiated claims in the manuscript that will require further contextualization, discussion, and most importantly, citation:

General comments:

I do understand and appreciate that the authors may consider English as a second language. However, there were points where the narrative was unclear and could benefit from copyediting. I was able to understand the thrust, but re-reading and re-evaluating readability will help make the manuscript clearer for the reader.

***Response:*** *The manuscript was additionally proofread, we hope that this now meets your expectations.*

The authors should refrain from using the term “confirmed” when referencing their own hypotheses or the results of extant literature. The nature of parametric statistics precludes the confirmation of results and only *support for* the observed results.

***Response:*** *The corresponding phrase (line 249) was changed to “Results support for…”. We additionally searched the manuscript for the term “confirm” and changed it when used inappropriately. Now the word confirmed is only used for other studies. We did not mean to confuse the reader or to give wrong information.*

Background

Generally, when introducing a cited study please briefly describe the sample and the design of the cited study. This will give the reader more context for the results being described

***Response:*** *Short information about the design and sample of the cited studies was added.*

Lines 73 – 74 will require a citation for the assertion that “research...has revealed that internal entrapment seems to be more important...”

***Response:*** *We are sorry, there was a reference missing. This information came from O'Connor & Portzky (2018). We added this citation/reference.*

Please briefly describe the findings of Owen, Dempsey et al., more in depth

***Response:*** *Information about the responding design and sample of this study was added, as well as its outcome distinguishing between internal and external entrapment.*

Lines 78 – 79 appears to be an assertion that comes out of the blue; however, it becomes clearer when discussing this assertion in the Discussion section of the manuscript, lines 230 – 233. Clarifying this assertion in the Introduction would be useful

***Response:*** *The explanations made in the discussion have been added to the Introduction. We hope everything is now easier to understand.*

Line 97; although I understand the reflex to argue from a perspective of reason, it is not sufficient to argue for the differential effects of entrapment dimensions on hopelessness based on reason; reasonable based on...? The Cry of Pain model? Johnson, Gooding? These at least should be cited and expanded upon a bit more

***Response:*** *The word “reasonable” was changed to “conceivable” to support the theoretical assertion which was made according to the empirical findings mentioned above. We also added that this assumption is based on the above mentioned studies and considerations.*

Measures

Please provide a detailed breakdown of demographics in a table to further contextualize the sample; among the variables already include, likely additions should include racial identity, gender identity, and the baseline risk variables (suicide attempt, suicidal ideation, etc.)

***Response:*** *We already give information on suicide attempt in the past as well as suicidal ideation and gender identity. However, we also added a table (Table 1 in supplementary material) including some more sociodemographic characteristics and risk variables as supplementary material. We hope that this meets your expectations. However, there is no information on racial identity; it is not common to assess this in Germany.*

Please provide further detail as to whether measures were sum scored or mean scored

***Response:*** *all measures were sum scored, which has been added to the descriptions.*

Please provide the following estimates in the correlation table in addition to those provided: standard error (SE), skew, kurtosis, range, for each of the constructs measured

***Response:*** *The missing information has been added to Table 1 in the manuscript.*

Statistical analyses

Line 165; the assertion of either full or partial mediation is contrary to the hypotheses and strikes of HARKing; please clarify here and in the hypotheses whether you predict full or partial mediation, not both.

***Response:*** *The part of the sentence in which the full mediation was mentioned was deleted to clarify our partial mediation hypothesis. We apologize for this inconvenience. We do not know how this happened; it might have happened during the translation process but we clearly did not conduct HARKing, which is quite a strong accusation. Our hypotheses were formulated before data collection even began.*

It seems reasonable that the authors should test the hypothesized model with the Entrapment Scale total score considering their intention to determine whether internal vs. external entrapment explain more variance in SI as explained by hopelessness; this would make for a potentially stronger paper by match some of their cited literature (i.e., Littlewood, Gooding), testing an inference of the IMV, and align with their assertion that “it remains unclear how and why internal and external entrapment influence suicidal ideation”. Testing all three models would permit more nuanced discussion of theoretical implications.

***Response:*** *Our intention above all was to investigate the difference between internal and external entrapment relating to suicidal ideation and hopelessness. The missing differentiation of internal and external entrapment in the IMV model was one of our motivations for this research. Along with our hypotheses, we expected the effect of internal entrapment to be greater. To examine our hypotheses it was necessary to conduct the models separately for internal and external entrapment. Due to your suggestions, we conducted post-hoc a third mediation model including total entrapment as a predictor. We added these post-hoc analyses to the statistical analyses section and provide the results in the supplementary material. All coefficients were significant (p < .001. Overall, when comparing the results of total entrapment to those of the preceding analyses, the completely standardized indirect effect of total entrapment was smaller than the completely standardized indirect effect of external entrapment (for more detailed results see supplementary material. This suggests that the role of external entrapment needs to be further examined independently of internal entrapment especially because despite the statistics the treatment differs (s. comment below).*

Discussion

Line 263; please compare and contrast how your findings are contrary to those of the cited studies

***Response:*** *Our findings are contrary to those of Höller et al. (2021) and Lucht et al. (2020), as our results show an effect of external entrapment on suicidal ideation. We added discussion on this topic to the manuscript.*

Lines 267 – 274; these are major assumptions regarding the impact of the COVID-19 pandemic on suicidal ideation and external entrapment that will require contextualization within the available literature. For example, Bryan, Bryan, and Baker (2021) found no relationship between physical distancing and with worse mental health outcomes. This observation has implications for the relationship between the COVID-19 pandemic and external entrapment.

***Response:*** *Thank you for this suggestion. Unfortunately, we cannot find the paper you mentioned and are therefore not exactly sure what you mean. If you could specify, we would be happy to adapt the manuscript accordingly. Physical distancing is not the same as feeling externally entrapped because of the governments’ social distancing rules. Feeling entrapped in this case does not necessarily mean to actually be trapped in the sense of physical distance but more the feeling of being trapped due to the rules and no hope that this will change any time in the near future. But we could further adapt the manuscript to the paper on physical distancing when we know which paper exactly you mean. We made some changes to weaken our conclusions and hope this meets your expectations now.*

Line 278; given that both external and internal entrapment in relation to suicidal ideation were significantly explained by hopelessness to some extent, I am not convinced that these constructs require separation.

***Response:*** *Both internal and external entrapment were mentioned here to emphasize the connection with suicidal ideation and hopelessness. We added one more sentences on the differentiation of internal and external entrapment. We are not sure whether we understand your point correctly. Statistically, internal and external entrapment are highly correlated but yet distinct constructs. In this study, the correlation between internal and external entrapment is high with .85. However, the entrapment scale was designed to assess both internal and external entrapment and study results that examined differences in internal and external entrapment found different associations of the constructs with suicidal ideation. There is a difference whether your patient has rumination (internal entrapment) or whether he/she is hopeless due to external circumstances (job loss, relationship problems 🡪 external entrapment). The two constructs measure different states that need different treatment approaches. Those treatment approaches have been discussed in the discussion section, too. The separation of these constructs is only a consequence of the mixed findings. Even if both were equally important in the development of suicidal ideation, the interventions would differ. For clinical practice and for suicide prevention, this needs to be considered. Both construcs are important in the development of suicidal ideation but it would be interesting to investigate how different treatment approaches could prevent individuals from developing suicidal ideation.*

Their conclusions may be supported by inclusion of the proposed total score model. If a worse model fit is observed in contrast to the internal or external entrapment models, this conclusion would be significantly strengthened

***Response:*** *We foresee from a third model since this was not our goal. Our goal was to examine differential effects from internal and external entrapment on suicidal ideation. Especially because this differentiation is especially important when talking about possible interventions see our comment above. However, we did follow your suggestion and added a third model to the manuscript. This is mentioned as post-hoc analysis in the statistical anylses section. Results can be found in the supplementary material.*

Additionally, the authors may wish to discuss the findings in different contexts; for example, they may wish to reference the findings of Oakey-Frost et al., 2021 who found that internal but not external entrapment with fearlessness about death explained the relationship between PTSD and suicidal ideation. Thus, the effect of one vs. the other may vary according to important interindividual differences

***Response:*** *These important findings were added to the manuscript. Thank you for this suggestion. However, we would like to mention, that, in our view, this underlines again the importance of differentiating between these constructs. Even if both constructs were equally important, the importance might differ depending on interindividual differences. Maybe there is no “one fits all”-model.*

:

Lines 279 – 278; the argument for differential treatment approaches for internal and external entrapment is potentially reasonable, but unsubstantiated with regard to approaches to external entrapment specifically.

***Response:*** *In fact, there are no evidence-based interventions yet. That is why we provide some theoretical considerations here. We added a sentence that those are only theoretical considerations and that more empirical research in this area is needed.*

Additionally, the question must be asked: are we aiming to improve entrapment or suicidal thoughts and behaviors? Other viable treatment options do exist and should be discussed; The Collaborative Assessment and Management of Suicidality (CAMS; Jobes, 2017) is a framework used to target entrapment OR internal vs. external entrapment as the driver of suicidal thoughts and behaviors based on the needs of the individual in question. This critique includes lines 311 – 312. Thus, the authors may be correct that internal vs. external entrapment may require different approaches based on the needs of the individual and their suicidal driver. But this claim has to be substantiated with citation and discussion.

***Response****: We are aware that there are intervention programs for patients regarding suicidality but as you said there is a difference of treating suicidality and focusing on proximal risk factors. Intervention targeting proximal risk factors would aim at preventing patients from even developing suicidal ideation. We added reference to the CAMS to the manuscript. We added discussion on this to the manuscript. We also added to the manuscript that we have to differentiate between programs that treat suicidality and interventions that target proximal risk factors. We hope that this meets your expectations now.*

Finally, the authors should discuss the limitations of cross-sectional mediation in the context of Maxwell & Cole, 2007.

***Response****: Maxwell, Cole and Mitchell (2011) was added to the limitations as this article discusses partial mediation models in cross-sectional studies. Thank you for this suggestion.*

Figure

I would prefer that arrows in the models are bidirectional. While the conclusions may be theoretically supported, inferring directionality and therefore temporal relationships is not supported in cross-sectional mediation models.

***Response****: We amended the mediation models but hope that this is not misleading.*

Thank you for allowing me to review your manuscript and for your valuable contribution to the suicide prevention literature base. I hope that my comments and feedback are of value to the authors and to the editor(s) of the journal. I genuinely appreciate the opportunity to engage in this valuable scientific discourse.

Höller, I., Rath, D., Teismann, T., Glaesmer, H., Lucht, L., Paashaus, L., Schönfelder, A., Juckel, G., & Forkmann, T. (2021). Defeat, entrapment, and suicidal ideation: Twelve‐month trajectories. *Suicide and Life‐Threatening Behavior*.

O’Connor, R. C., & Portzky, G. (2018). The relationship between entrapment and suicidal behavior through the lens of the integrated motivational–volitional model of suicidal behavior. *Curr Opin Psychol, 22*, 12-17. <https://doi.org/10.1016/j.copsyc.2017.07.021>

Owen, R., Dempsey, R., Jones, S., & Gooding, P. (2018). Defeat and entrapment in bipolar disorder: exploring the relationship with suicidal ideation from a psychological theoretical perspective. *Suicide and Life‐Threatening Behavior, 48*(1), 116-128. <https://doi.org/10.1111/sltb.12343>

Rasmussen, S. A., Fraser, L., Gotz, M., MacHale, S., Mackie, R., Masterton, G., McConachie, S., & O'Connor, R. C. (2010, 3/2010). Elaborating the cry of pain model of suicidality: Testing a psychological model in a sample of first-time and repeat self-harm patients. *British Journal of Clinical Psychology, 49*(1), 15-30. <https://doi.org/10.1348/014466509X415735>
