# Supplementary material for: Trapped in my inner prison—Cross-sectional examination of internal and external entrapment, hopelessness and suicidal ideation
Source: PLoS One. 2022 Jul 19;17(7):e0270985. doi: 10.1371/journal.pone.0270985 (PMC9295950; doi:10.1371/journal.pone.0270985)
Supplement: Attachment — Submitted filename: DetailedResponsetotheReviewes_Revision_Nr_2.docx [file pone.0270985.s005.docx]

**Reviewer #1**

Many thanks to the authors for their thoughtful responses to the feedback. I have just a few more comments that I think would be best addressed prior to publication:

Background

Line 61, 64, and 67 use the word “confirm[ed]” in relation to the results of the cited studies. I don’t want to sound like a broken record, but “confirming” entrapment as a risk factor in a cross-sectional mediation analysis is a mischaracterization. I think it is incredibly important to not overstate the implications of any results, especially to the general public.

***Response:*** *We are sorry that you got the impression we wanted to overstate the findings. To not overrate the results of the cited study the term “confirmed” was changed into “shown” (line 61), “pointed to” (line 64) and “demonstrated” (line 67/70).*

Discussion

In previous feedback, the authors indicated their willingness to discuss differential findings with relation to social distancing and external entrapment in the context of the COVID-19 pandemic. Unfortunately, I provided the wrong year for the citation I included. Would the authors be willing to Discuss their findings from Lines 287 – 297 in the context of the following citation:
Bryan, Bryan, & Baker. (2020). Associations among state-level physical distancing measures and suicidal thoughts and behaviors among U.S. adults during the early COVID-19 pandemic. Suicide and Life-Threatening Behavior.

***Response:*** *This reference was added to the manuscript (lines 294-300). We discussed the findings of Bryan et al. (2020) that there were no correlations between physical distancing and suicidal ideation, however, as the authors state themselves, they collected data at the very beginning of the pandemic, while we collected data when the pandemic was already going on for more than a year.*

To first put the following comment in context, I apologize if my previous feedback was not clearly articulated. I want to be clearer about where I am coming from:

Line 310, their assertion that “there have been no evidence-based interventions for the reduction of entrapment” is where I would recommend, they discuss CAMS. CAMS targets suicidal ideation and suicidal behavior via conceptualization of the patient’s “drivers” which indeed may include perceptions of internal entrapment and/or external entrapment. The clinician then uses their knowledge to recommend particular treatment orientations to target the driver. Thus, treating suicidal ideation occurs via treatment of the factors that lead to suicidal ideation. Additionally, they argue that CAMS and ASSIP do not target proximal risk; from a CAMS perspective, it does. The first session of CAMS includes collaborative formulation of a Safety/Crisis Prevention Plan; these interventions are inherently designed to target “proximal risk” (Line 323).

***Response:*** *CAMS is now named to be an effective treatment that is addressing internal and/or external entrapment. We extended the paragraph on CAMS starting on line 334-340. We hope that this meets your expectations.*

In essence, I agree with the authors: internal vs. external entrapment may be differentiated based on their treatment needs. I also believe the authors have a good opportunity to make their argument in the context of available interventions. Treatment may be accomplished via CAMS which, again, identifies a driver (e.g., internal entrapment) and then uses patient specific interventions to target the driver (e.g., cognitive therapy [see Jobes, 2017]).

***Response:*** *We agree with you that is why we added CAMS as a possible treatment (See comment above)*

Overall, I genuinely appreciate the author’s responses and persistence thus far. Thank you again for the opportunity to review your manuscript.

**Reviewer #2**

The authors have responded to comments and improved the paper. Just some minor comments remain.

Methods
The Sample results (p.5, l. 105-112) should be reported in the Results section.
Response: We consider this sample information necessary to provide insight into the composition of the sample at this point. The sample information is not reported in the results section because the results section should mainly highlight the results of the statistical analyses that were conducted to test our hypotheses (i.e., correlation and mediation analysis). We hope that it is okay if we leave it like that.
Reviewer response: The standard is to report the details of the collected sample in the Results, rather than the Methods.

***Response:*** *We think that this actually depends on the journal but we understand where you are coming from so we followed your suggestion. The information of the sample results is now reported in the Results section. The sub-headlines “Correlation Analysis” and “Mediation Analysis” have been added to the Results section to obtain a better structure.*

Measures
How were past and current mental disorders measured?
Response: Participants were asked with single items each whether they had a mental disorder in the past or at the moment and whether they received treatment. We are aware that this is only self-report, this is why we used the wording “participants reported a mental disorder”. We added the self-report nature to the limitations.
Reviewer response: The details about this question should be included in the Measures section.

***Response:*** *The information delivered above was added to the Measures section.*


The scoring of the BSS is not clearly explained (sentence on p.6, l.136-137). How were the five items
which were used scored?
Response: The (total) BSS score ranges from 0 to 38 and is formed from the sum of the first 19 items. The last two items (20 and 21) describe former suicide attempts (and not thoughts) and are usually not included in the total score. However, because of the ambiguous factor structure of the total BSS score, the BSS-Screen was used to describe suicidal ideation. In contrast to the total BSS-Score, the BSS-Screen score has a unidimensional factor structure. This BSS-Screen includes the sum score of only the first five items of the BSS. We added information on this to the manuscript.
Reviewer response: The sentence on p.7, l. 148-149 of the revised manuscript is still unclear English and needs editing: “The first 19 items are summed up in case items 4 and 5 have been answered at least 1.”

***Response:*** *We are sorry the reformulation did not meet your expectation. We tried again and also asked an independent person whether it is understandable how the sum score was build. The sentences now read like this in the manuscript:* *When participants answered items 4 and 5 (screening items) with at least 1, the first 19 items were used to build a sum score. Otherwise, participants had a sum score of zero.*

In addition to the above points, a further limitation of the study is that respondents tended to be predominantly young, and perhaps with a large proportion of university and tertiary educated persons, although this will depend on what the standard for Germany is. A brief comment on how the sample compares to the German population may be useful.

***Response:*** *Information of the German population was added to the Strengths and Limitations – Section (lines 347-351). We emphasized that our sample includes a higher female percentage, is younger, and a higher percentage of high education than the general German population. We also stated in the limitation section that this limits the generalizability of our findings.*

Another comment I have is that internal and external entrapment are so highly related that they would likely often occur together and may not be always easy to separate or be treated separately in psychological interventions.

***Response:*** *Indeed, internal entrapment and external entrapment are highly related and often occur together. We modified the discussion and emphasize that it might not be easy to separate them in clinical practice and added more to the treatment program CAMS that targets both internal and external entrapment.*
